# CEA expression heterogeneity and plasticity confer resistance to the CEA-targeting bispecific immunotherapy antibody cibisatamab (CEA-TCB) in patient-derived colorectal cancer organoids

**DOI:** 10.1186/s40425-019-0575-3

**Published:** 2019-04-15

**Authors:** Reyes Gonzalez-Exposito, Maria Semiannikova, Beatrice Griffiths, Khurum Khan, Louise J. Barber, Andrew Woolston, Georgia Spain, Katharina von Loga, Ben Challoner, Radhika Patel, Michael Ranes, Amanda Swain, Janet Thomas, Annette Bryant, Claire Saffery, Nicos Fotiadis, Sebastian Guettler, David Mansfield, Alan Melcher, Thomas Powles, Sheela Rao, David Watkins, Ian Chau, Nik Matthews, Fredrik Wallberg, Naureen Starling, David Cunningham, Marco Gerlinger

**Affiliations:** 1 Translational Oncogenomics Laboratory, Centre for Evolution and Cancer, The Institute of Cancer Research, 237 Fulham Road, London, SW3 6JB UK; 2 Gastrointestinal Cancer Unit, The Royal Marsden NHS Foundation Trust, London and Sutton, UK; 3 Flow Cytometry and Light Microscopy Core Facility, The Institute of Cancer Research, London, UK; 4 Structural Biology of Cell Signalling Laboratory, The Institute of Cancer Research, London, UK; 5 Tumour Profiling Unit, The Institute of Cancer Research, London, UK; 6 Department of Radiology, The Royal Marsden NHS Foundation Trust, London and Sutton, UK; 7 Translational Immunotherapy Laboratory, The Institute of Cancer Research, London, UK; 8 Barts Cancer Institute, Queen Mary University, London, UK

**Keywords:** Cibisatamab, CEA, Immunotherapy, Patient-derived organoids, WNT/β-catenin

## Abstract

**Background:**

The T cell bispecific antibody cibisatamab (CEA-TCB) binds Carcino-Embryonic Antigen (CEA) on cancer cells and CD3 on T cells, which triggers T cell killing of cancer cell lines expressing moderate to high levels of CEA at the cell surface. Patient derived colorectal cancer organoids (PDOs) may more accurately represent patient tumors than established cell lines which potentially enables more detailed insights into mechanisms of cibisatamab resistance and sensitivity.

**Methods:**

We established PDOs from multidrug-resistant metastatic CRCs. CEA expression of PDOs was determined by FACS and sensitivity to cibisatamab immunotherapy was assessed by co-culture of PDOs and allogeneic CD8 T cells.

**Results:**

PDOs could be categorized into 3 groups based on CEA cell-surface expression: CEA_hi_ (*n* = 3), CEA_lo_ (*n* = 1) and CEA_mixed_ PDOs (*n* = 4), that stably maintained populations of CEA_hi_ and CEA_lo_ cells, which has not previously been described in CRC cell lines. CEA_hi_ PDOs were sensitive whereas CEA_lo_ PDOs showed resistance to cibisatamab. PDOs with mixed expression showed low sensitivity to cibisatamab, suggesting that CEA_lo_ cells maintain cancer cell growth. Culture of FACS-sorted CEA_hi_ and CEA_lo_ cells from PDOs with mixed CEA expression demonstrated high plasticity of CEA expression, contributing to resistance acquisition through CEA antigen loss. RNA-sequencing revealed increased WNT/β-catenin pathway activity in CEA_lo_ cells. Cell surface CEA expression was up-regulated by inhibitors of the WNT/β-catenin pathway.

**Conclusions:**

Based on these preclinical findings, heterogeneity and plasticity of CEA expression appear to confer low cibisatamab sensitivity in PDOs, supporting further clinical evaluation of their predictive effect in CRC. Pharmacological inhibition of the WNT/β-catenin pathway may be a rational combination to sensitize CRCs to cibisatamab. Our novel PDO and T cell co-culture immunotherapy models enable pre-clinical discovery of candidate biomarkers and combination therapies that may inform and accelerate the development of immuno-oncology agents in the clinic.

**Electronic supplementary material:**

The online version of this article (10.1186/s40425-019-0575-3) contains supplementary material, which is available to authorized users.

## Background

Colorectal cancer (CRC) is the third most common cause of cancer related mortality worldwide [1]. Median overall survival for patients with metastatic CRC has increased to over 24 months since the introduction of anti-vascular endothelial growth factor (VEGF) and anti-epidermal growth factor (EGFR) targeted therapies [2]. Trifluridine-tipiracil (TAS102) and the multi-kinase inhibitor regorafenib are recently developed third line therapy options but their survival benefit is only modest [3, 4]. Thus, new therapies are needed to further improve outcomes of patients with metastatic CRC.

Checkpoint-inhibiting immunotherapies recently led to substantial increase in survival in some tumor types. These include hypermutated microsatellite instable (MSI) CRCs [5, 6] but checkpoint inhibitors showed no activity in microsatellite stable (MSS) CRCs. High numbers of mutation encoded neo-antigens in MSI CRCs are thought to foster spontaneous immune response with increased cytotoxic CD8 T cell infiltrates that are kept in check by cancer cells through upregulation of PD-L1 [7]. Unfortunately, only ~ 5% of metastatic CRCs display the MSI phenotype [8]. Thus, benefit from checkpoint inhibitors is currently restricted to a small subgroup of CRC patients [9].

The T cell bispecific antibody cibisatamab (CEA-TCB) is a novel immunotherapy that redirects T cells independently of their T cell receptor specificity to tumor cells expressing the carcinoembryonic antigen (CEA) glycoprotein at the cell surface [10]. A major advantage of T cell redirecting bispecific antibodies is that they mediate cancer cell recognition by T cells independently of neoantigen load. CEA is overexpressed on the cell surface of many colorectal cancers and cibisatamab is hence a promising immunotherapy agent for non hypermutated MSS CRCs.

Cibisatamab has a single binding site for the CD3 epsilon chain on T cells and two CEA binding sites which tune the binding avidity to cancer cells with moderate to high CEA cell surface expression [11]. This avoids targeting of healthy epithelial cells with low CEA expression levels, which are physiologically present in some tissues. Binding of cibisatamab to CEA on the surface of cancer cells and of CD3 on T cells triggers T cell activation, cytokine secretion and cytotoxic granule release. The phase I trial of cibisatamab in patients with CEA expressing metastatic CRCs that had failed at least two prior chemotherapy regimens showed antitumor activity with radiological shrinkage in 11% (4/36) and 50% (5/10) of patients treated with monotherapy or in combination with PD-L1-inhibiting antibodies, respectively [12, 13]. Based on these results, CEA is one of the most promising target antigens for immunotherapy in MSS CRCs. Although some patients in this dose escalation trial were treated with a dose below the final recommended dose, the response rates nevertheless indicate that a subgroup of tumors is resistant to treatment.

Molecular mechanisms of cibisatamab activity have been investigated in CRC cell lines in vitro using killing assays with peripheral blood mononuclear cells [11]. This identified CEA expression as a major determinant of cibisatamab sensitivity as only cell lines expressing moderate to high CEA levels were susceptible to T cell mediated killing. In contrast, cibisatamab sensitivity was independent of the presence of mutations in CRC driver genes such as *APC*, *TP53*, *KRAS* and *BRAF*.

Recently developed protocols allow the expansion and long term propagation of cancer cells as so called patient derived organoids (PDOs) from CRC biopsies [14]. PDOs have been suggested to better represent the biological characteristics of patient tumors than cancer cell lines which were often established decades ago and have undergone changes during long term culture on plastic. The ability to rapidly generate model systems from patients furthermore enables matching of disease stage and prior treatment history to those of patients in whom novel drugs are clinically tested.

Identification of resistance mechanisms for novel immunotherapies in patient-relevant preclinical models should define candidate biomarkers for the identification of sensitive patient subgroups or of combination therapy strategies that enhance efficacy by co-targeting of resistance pathways. This could maximize trial success rate and prevent drug attrition, which remains common for new cancer drugs in the absence of biomarkers. The aim of this study was to investigate mechanisms of resistance to cibisatamab immunotherapy beyond those identified in cell lines by using PDO models from treatment refractory metastatic CRCs. To this end, we established seven PDOs from chemotherapy resistant metastatic CRCs and one from a treatment naïve primary CRC and developed an in vitro co-culture assay with CD8 T cells to assess cibisatamab efficacy.

## Material and methods

### Human samples and cell lines

Imaging-guided core biopsies from metastatic colorectal cancers that had been treated with at least two prior lines of chemotherapy were obtained from the Prospect C and Prospect R trials (Chief investigator: D. Cunningham, UK national ethics committee approval numbers: 12/LO/0914 and 14/LO/1812, respectively). One endoscopic biopsy from a treatment naïve primary colorectal cancer was obtained from the FOrMAT trial (Chief investigator: N. Starling, UK national ethics committee approval number 13/LO/1274). Trials were run at the Royal Marsden Hospital and all patients provided written informed consent before trial inclusion. Anonymized buffy coats from healthy donors were obtained from the local blood bank (National ethics committee approval number 06/Q1206/106) or through the Improving Outcomes in Cancer biobanking protocol at the Barts Cancer Institute (Chief investigator: T. Powles, UK national ethics committee approval number: 13/EM/0327) from individuals providing written informed consent. DLD-1 and MKN-45 cell lines were obtained from the American Type Culture Collection and were maintained in RPMI 1640 medium supplemented with 10% FBS, 1X Glutamax and 100units/ml penicillin/streptomicin (Thermo Fisher).

### Generation of patient derived organoids

PDO cultures from CRC-01, CRC-02 and CRC-06 were established directly from core biopsies by rough chopping followed by embedding in growth factor reduced Matrigel (Corning). Very small biopsy fragments were available from CRC-03, CRC-04, CRC-05, CRC-07 and CRC-08 and these were first grafted subcutaneously or under the kidney capsule of female CD1 nude mice by the Tumour Profiling Unit at the Institute of Cancer Research (Home office licence number PD498FF8D). Mice were culled once tumors had grown and tumors were removed and dissociated in a gentleMAX Octo dissociator using the Human Tumour Dissociation Kit (Miltenyi Biotec). Mouse cells were magnetically removed using the Mouse Cell Depletion Kit (Miltenyi Biotec), and purified human tumour cells were embedded into growth factor reduced Matrigel. PDOs were expanded in Matrigel as described [14] using Advanced DMEM/F12 media supplemented with 1X Glutamax, 100 units/ml penicillin/streptomycin, 1X B27, 1X N2, 10mM HEPES (all Thermo Fisher), 1mM N-acetyl cysteine, 10mM nicotinamide, 10uM SB202190, 10nM gastrin, 10uM Y27632 (Sigma Aldrich), 10nM prostaglandin E2, 500nM A-83-01, 100ng/ml Wnt3a (Biotechne), 50ng/ml EGF (Merck), 1μg/ml R-Spondin, 100ng/ml Noggin, and 100ng/ml FGF10 (Peprotech). After at least 2 months of continuous growth in the matrigel matrix (minimum of 12 passages), the PDOs were first eGFP tagged (see below) and then adapted to grow in DMEM/F12 (Sigma Aldrich) with 20% fetal bovine serum (FBS), 1X Glutamax, 100 units/ml penicillin/streptomycin containing 2% Matrigel. PDO cultures were maintained in these conditions and used as required for T cell co-culture assays and FACS analysis. Genetic analyses of colon cancer driver genes were performed on each PDO line and these were identical to the mutations that had been identified in the matched tumor biopsies.

### Labelling of PDOs with nuclear eGFP

The nuclei of PDOs were labelled by introducing an eGFP tagged histone 2B construct (pLKO.1-LV-H2B-GFP) [15] to enable cell quantification by automated microscopy. For virus generation, HEK-293T cells were cultured in DMEM supplemented with 10%FBS, 1XGlutamax and 100units/ml penicillin/streptomycin. Lentiviral particles were produced by overnight transfection with a plasmid mixture containing 9 μg of pLKO.1-LV-H2B-GFP, 2.25 μg of psPAX2 packaging plasmid (gift from Didier Trono; Addgene plasmid #12260; http://n2t.net/addgene:12260; RRID:Addgene_12,260) and 0.75 μg of pMD2.G envelope plasmid (gift from Didier Trono; Addgene plasmid # 12259; http://n2t.net/addgene:12259; RRID:Addgene_12,259) using TransIT-293 transfection reagent (Mirus). The cells were media changed the following day, virus harvested after 24 h and passed through a 0.45uM filter before use. For lentiviral transduction PDOs were harvested from the cultures in Matrigel and dissociated to single cells using TrypLE Express (Thermo Fisher), and pelleted. The pellets were resuspended in media with the addition of virus and 1nM polybrene (Sigma Aldrich) and centrifuged at 300g for 1 h. The samples were resuspended and plated in culture for between 6 h and overnight, before replacing the media. Following recovery and expansion, eGFP positive cells were sorted by flow cytometry and further expanded before use.

### Surface CEA expression analysis by flow cytometry

Cell lines were harvested using enzyme-free Cell Dissociation Buffer (Thermo Fisher) and PDOs with TrypLE Express (Gibco). 2 × 10^5^ cells were stained with 20nM of the human anti-human CEA antibody CH1A1A (Roche) and 25μg/ml of the R-Phycoerythrin conjugated secondary antibody AffiniPure F(ab’)2 Fragment Goat Anti-Human IgG, Fcγ Fragment Specific (Stratech). DRAQ7 (Biostatus) staining was included for dead cell exclusion. CEA expression was analysed on a Sony SH800 flow cytometer. Gate boundaries were set at the trough between high and low CEA populations in PDOs with mixed CEA expression and identical gates were used across all samples. The percentage of CEA_hi_ and CEA_lo_ populations and mean fluorescence intensities (MFI) were calculated for each PDO.

### CEA expression analysis in human protein atlas samples

Microscopic images of 11 CRC samples stained by the Human Protein Atlas (www.proteinatlas.org) with the rabbit polyclonal antibody HPA019758 (Sigma Aldrich) which has been validated against RNA expression data, immunofluorescence staining with an independent antibody and by Western blot were downloaded and assessed for expression heterogeneity by two pathologists (K. von Loga and B. Challoner).

### CD8 T cells expansion from peripheral blood mononuclear cells

Peripheral Blood Mononuclear Cells (PBMCs) were isolated from buffy coats with Ficoll-Paque according to the manufacturer’s protocol (GE Healthcare). CD8 T cells were isolated from PBMCs with Human CD8 Dynabeads FlowComp (Thermo Fisher). The purity of CD8 T cells was assessed by flow cytometry (Alexa Fluor 488 anti-human CD8, Sony Biotechnology) and only populations with at least 90% CD8 positive cells were used for expansion with the CD3/CD28 Dynabeads Human T-Activator kit (Thermo Fisher) in RPMI 1640 supplemented with 10% FBS (Biosera), 1X Glutamax, 100units penicillin/streptomycin and 30 U/mL IL-2 (Sigma Aldrich) following the manufacturer’s protocol.

### Co-culture of PDOs and CD8 T cells

PDOs were harvested with TrypLE Express and neutralised with DMEM/F12 Ham medium (Sigma Aldrich) with 10% FBS. Cells were filtered through a 70um filter, counted and re-suspended in phenol-red free RPMI medium (Thermo Fisher) supplemented with 10% FBS (Biosera), 1XGlutamax and 100units penicillin-streptomycin. On day 0, 5000 tumor cells per well of a 96 well-plate (Corning Special Optics Microplate) were plated. CD8 T cells were added on day 1 at the indicated effector to target (E:T) ratios with 20nM of cibisatamab or 20nM of the untargeted negative control antibody DP47-TCB (both provided by Roche). Tumor cells without CD8 T cells and without antibody were also included as controls. All conditions were plated in triplicates and at least 3 different healthy donors were tested on each of the 8 PDOs.

### Cancer cell growth assessment by immunofluorescence microscopy

The GFP confluence was quantified every 48h–72h over a 10-day period using the GFP confluence application on the Celigo Imaging Cytometer (Nexcelom Bioscience). GFP confluence analysis was able to track the growth of GFP positive PDO cells over multiple timepoints without erroneously counting the T cells in the co-culture. Confluence analysis was furthermore superior to the counting of cell nuclei which generated inaccurate results in areas of high cancer cell density such as the PDO centre. The main advantage of confluence analysis over measuring spheroid diameters is the ability to track even the the growth of PDOs showing highly variable shapes. Growth curves were generated with CD8 T cells from three different healthy blood donors. The percentage growth reduction was calculated from readings taken between days 7 to 9, before PDOs showed growth retardation, likely due to exhaustion of the growth media. In order to calculate the percentage of growth reduction, confluence at day 1 was subtracted and the confluence in wells treated with the DP47-TCB control antibody at the endpoint was set to 100%.

### Flow sorting of CEA_hi_ and CEA_lo_ cells

PDOs cells were harvested with TrypLE Express, filtered through a 70um filter to obtain a single cell solution and 2 × 10^6^ cells were stained with DAPI for dead cell exclusion, 20nM of the CH1A1A antibody (Roche) and 25μg/ml of the R-Phycoerythrin conjugated secondary antibody (Stratech) and sorted in CEA_hi_ and CEA_lo_ cells in an ARIAIII cytometer (BD Biosciences) in the FACS core facility at the Institute of Cancer Research. Cells were collected in cold DMEM supplemented with 10% FBS and pellets were frozen at -80°C after centrifugation for RNA-sequencing. CEA_hi_ and CEA_lo_ cells sorted from CRC-06 and CRC-08 were also grown for 1 month in DMEM/F12 Ham medium with 20% FBS, penicillin/streptomycin and 2% Matrigel.

### Gene expression analysis

Total RNA was extracted from frozen pellets of flow sorted of CEA_hi_ and CEA_lo_ populations from CRC-03, CRC-06 and CRC-08 using RNAeasy Plus (Qiagen) and quantified with Qubit RNA High Sensitivity assay (Thermo Fisher). RNA sequencing libraries were prepared using the QuantSeq 3′-mRNA-Seq Library Prep Kit FWD for Illumina (Lexogen) using the manufacturer’s protocol. A minimum of 5 ng input RNA was used from each sample and qPCR was performed on all samples before PCR amplification of the library to determine optimal cycle number following the manufacturer’s recommendations. Final libraries were quantified in duplicate using Qubit-HS and Bioanalyzer High Sensitivity (Agilent) before pooling. Libraries were sequenced with 100 single read cycles on an Illumina HiSeq2500 in rapid mode. Alignment and gene counting were performed on the Lexogen QuantSeq data analysis pipeline on the Bluebee cloud. Annotation data on the GRCh38.p12 genome assembly was exported from Ensembl with Biomart. The ID’s of protein coding genes were identified using the gtf file filtered on the ‘gene_biotype’ field. The Quantseq raw count data were then filtered for this gene set and the Ensembl ID’s converted to gene symbol using the Homo.sapiens Bioconductor package. Lowly expressed genes containing fewer than 10 counts were removed from the dataset. The count data were then normalised using the estimateSizeFactors and counts functions from the DESeq2 Bioconductor package [16].

### WNT/β-catenin pathway inhibition assay

10^5^ PDO cells/well were seeded in 12 well plates and allowed to attach overnight. Media were changed and cells were treated with DMSO control or with 10uM tankyrase inhibitor (Compound 21, kindly provided by Prof Lord) [17] or 10uM porcupine inhibitor (LGK-974, SelleckChem) for 3 days. Cells were harvested using TrypLE Express, stained for CEA with the CH1A1A primary antibody and the R-Phycoerythrin conjugated secondary antibody and analyzed by FACS as described above.

### Statistical analyses

Pearson correlation analysis and the paired t-tests were performed with the GraphPad Prism software. All *p* values are two tailed. Gene set enrichment analysis was performed with the GSEA software V3.0 using 5000 gene set permutations and the Hallmarks V6.2 gene set collection [18].

## Results

### Generation of patient derived organoids from colorectal cancers

CRC PDOs were established as 3D cultures in Matrigel a) directly from core biopsies from three chemotherapy resistant metastatic CRCs (CRC-01, CRC-02, CRC-06), b) from small core biopsies of four chemotherapy resistant metastatic CRCs which were first expanded as xenografts in immunodeficient mice (CRC-03, CRC-04, CRC-05, CRC-07) and c) from an endoscopic biopsy taken from a treatment naive primary CRC (CRC-08). Each PDO was continuously grown for at least 2 months in Matrigel to test for long term viability. They were labelled with a lentivirus encoding a histone tagged nuclear enhanced green fluorescent protein (eGFP) and were subsequently transferred into culture conditions with 2% Matrigel dissolved in growth medium. Matrigel does not form a solid culture matrix at this dilution and allows PDOs to attach to the bottom of the plastic plate. These culture conditions facilitate interaction with T cells and allow monitoring of PDO growth with wide field fluorescence light microscopy.

### CEA expression heterogeneity in patient derived CRC organoids

**Fig. 1:**
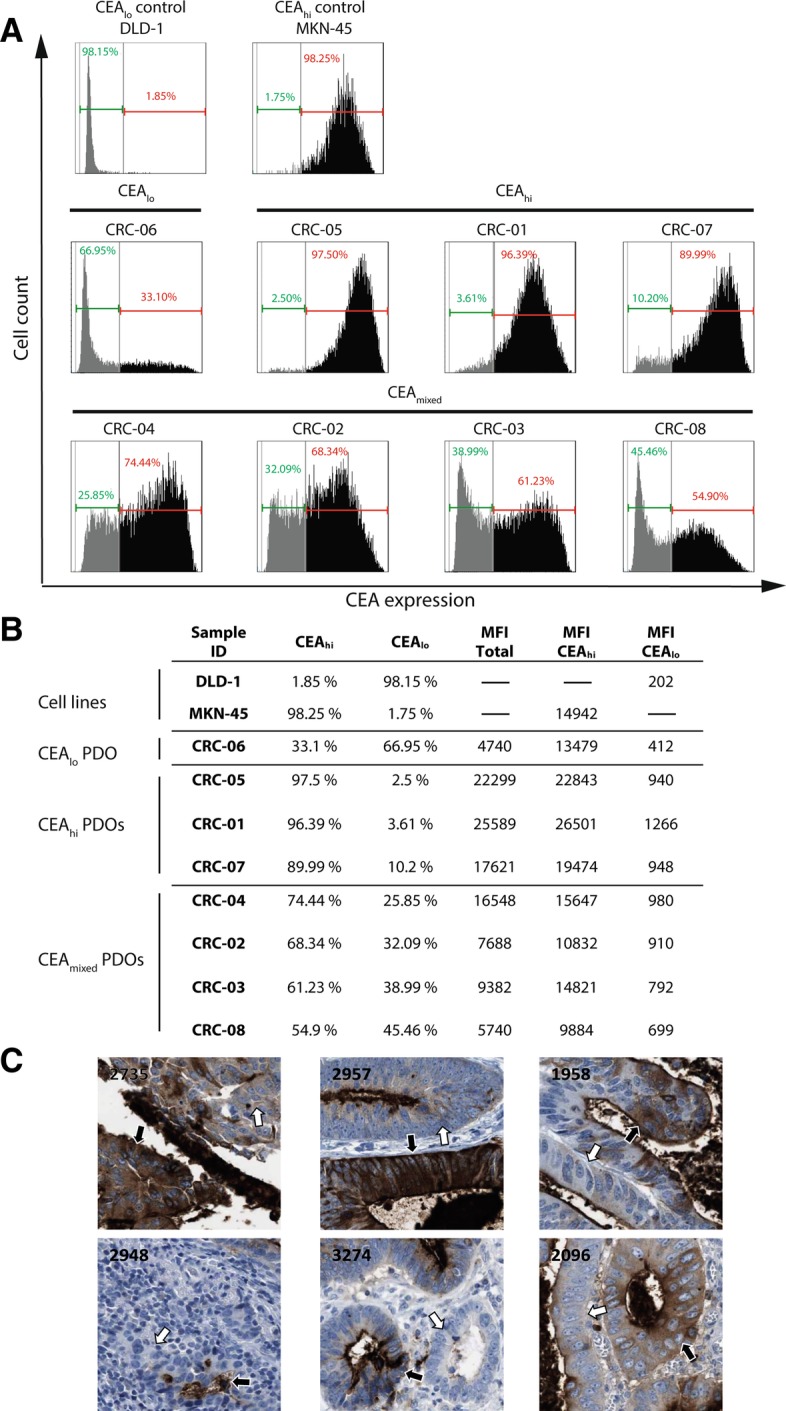
**a**: FACS analysis of CEA cell surface expression for DLD-1 and MKN-45 cell lines and 8 PDOs. Gates were adjusted on the trough of CRC-03 and identical gates were used to quantify the percentage of CEA_hi/lo_ cells in all lines. **b**: Summary of CEA _hi/lo_ percentages and measured mean fluorescent intensities (MFIs) of the data in panel A. **c**: CEA protein expression heterogeneity identified in 6/11 CRC samples stained with the anti-CEA/CEACAM5 antibody HPA019758. Examples of CEA heterogeneity are highlighted by white (low CEA) and black (high CEA) arrows. Numbers indicate the Human Protein Atlas patient IDs. (images: courtesy of the Human Protein Atlas v18.proteinatlas.org; link: https://www.proteinatlas.org/ENSG00000105388-CEACAM5/pathology/tissue/colorectal+cancer)

PDOs were dissociated into a single cell suspension and CEA cell surface expression was analysed by FACS using the CH1A1A antibody which has identical CEA antigen binding sites to the cibisatamab bispecific antibody (Fig. 1a). The DLD-1 cell line which had very low CEA surface expression and the MKN-45 cell line which was most strongly positive among 110 previously tested cell lines were included as controls [11]. Three of the PDOs showed high CEA expression (CRC-05, CRC-01 and CRC-07) with MFI values exceeding those of the MKN-45 positive control (Fig. 1b). A small fraction of cells (2.5–10.2% of the whole population) with low CEA expression were also detected in each of these PDOs. CEA expression was predominantly negative in one PDO (CRC-06) but this also showed CEA expression heterogeneity based on the presence of a subpopulation with high CEA expression (33.1% of the whole population). Similar heterogeneity of CEA expression was not observed in the DLD-1 and MKN-45 cell lines.

The most striking CEA expression heterogeneity was detected in 4 PDOs (CRC-02, CRC-03, CRC-04 and CRC-08) which each contained large subpopulations of CEA_hi_ and CEA_lo_ cells. The MFI of the CEA_hi_ subpopulations were similar to MKN-45 in two PDOs (CRC-03, CRC-04) and moderately lower in two others (CRC-02, CRC-08). A proportion of the CEA_lo_ cells in each of these four PDOs showed CEA expression levels which were as low as in the DLD-1 cell line, demonstrating heterogeneity across a broad range of CEA expression levels.

The heterogeneous CEA expression profiles of these PDOs is reminiscent of the CEA expression heterogeneity which has been described in CRC samples from patients [19]. We furthermore evaluated CRC tissue samples that had been stained with a validated CEA antibody by the Human Protein Atlas [20] and this also revealed CEA expression heterogeneity in 6/11 samples (54%; Fig. 1c). Importantly, similar expression heterogeneity to that observed in PDO samples was not present within DLD1 or MKN-45 cells and bimodal expression profiles have, to our knowledge, not previously been described in CRC cell lines. This supports the notion that PDOs better represent the molecular heterogeneity of colorectal cancers than established cell lines.

### Cibisatamab sensitivity of PDOs in an allogeneic T cell co-culture assay

**Fig. 2:**
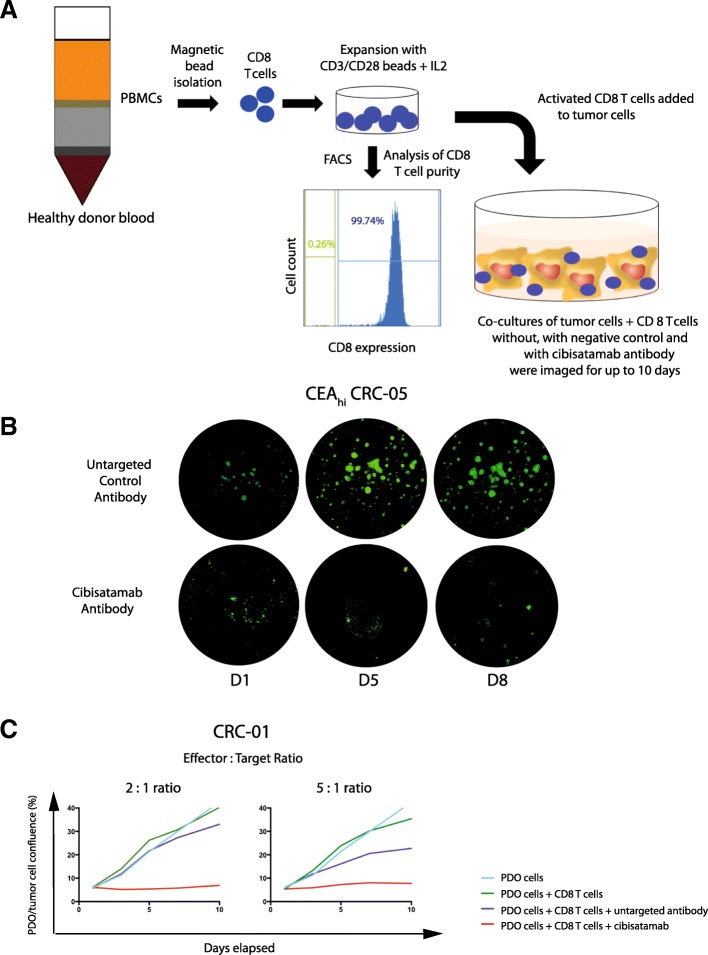
**a**: Diagram of the PDO and allogeneic CD8 T cell co-culture model. **b**: Example showing GFP fluorescence imaging of a co-culture model treated with the bispecific untargeted control antibody and cibisatamab over a period of 8 days. **c**: Growth curves generated with different effector to target (E:T) ratios in the CEA_hi_ PDO CRC-01 during assay development

In order to assess the sensitivity of PDOs to cibisatamab immunotherapy, CD8 T cells were isolated from allogeneic healthy donor peripheral blood mononuclear cells (PBMCs) by magnetic bead sorting and expanded in vitro with IL2 and CD3/CD28 beads for 7–14 days. GFP-tagged CRC PDO cells were then seeded in 96 well plates, T cells were added the following day (Fig. 2a) and the co-cultures were imaged every 2–3 days on an automated 96 well plate fluorescence microscope (Fig. 2b). Effector to target (E:T) ratios of 2:1 and 5:1 were tested and an E:T of 2:1 was chosen for subsequent experiments as it effectively suppressed growth of the CEA_hi_ PDO CRC-01 and showed no activity in the presence of the untargeted TCB antibody (DP47-TCB) which was used as a negative control (Fig. 2c). Co-culture with CD8 T cells without any antibody was included as a further control to enable the identification of alloreactive donor T cells. Co-cultures in which T cells showed alloreactivity (observed in less than one in ten experiments) were excluded from the analysis and assays were repeated until each PDO line was tested with CD8 T cells from 3 independent allogeneic donors.

**Fig. 3:**
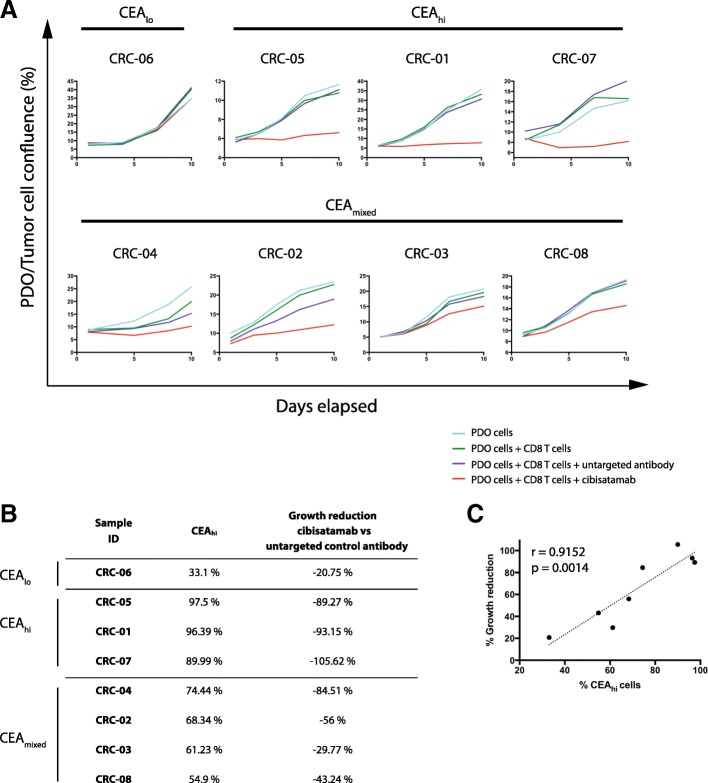
**a**: Growth curves for all eight PDO lines treated with cibisatamab or controls during 10 days of co-culture. Each PDO was cultured with T cells from three different allogeneic donors at an E:T ratio of 2:1 and means are shown. **b**: Comparison of the fraction of CEA_hi_ cells in each PDO with the growth reduction in achieved at the essay endpoint in panel A. **c**: Correlation analysis of growth reduction and the fraction of CEA_hi_ cells for all PDOs. A linear regression line and the Pearson correlation coefficient and *p* value of the significance test are shown

All three CEA_hi_ PDOs were highly sensitive to treatment with CD8 T cells and cibisatamab whereas the CEA_lo_ PDO CRC-06 showed resistance under these experimental conditions, as anticipated (Fig. 3a). Compared to conventional T cell killing assays with cibisatamab which showed fractional killing of ~ 40–50% of CEA_hi_ cancer cell lines after 24–48 h [11], our assay assesses the impact over a period of 7–10 days and this showed 89–100% growth inhibition in CEA_hi_ PDOs. This confirms the high efficacy of cibisatamab to re-direct T cells to antigen positive cells in this assay.

We next tested the four PDOs with mixed CEA expression. Each of these continued to proliferate despite treatment with cibisatamab and T cells, with only a moderate reduction of the cancer cell growth rate compared to controls (Fig. 3a and b). Thus, PDOs with mixed CEA expression only showed a partial response to this CEA targeting immunotherapy. Correlation analysis of the achieved growth reduction with the fraction of CEA_hi_ cancer cells in each PDO showed a strong and significant correlation (r = 0.9152, 95% CI: 0.593 to 0.9848; *p* = 0.0014; Fig. 3c). This substantiates that only the CEA_hi_ population is sensitive whereas CEA_lo_ cells continue to grow and promote resistance.

Taken together, CEA expression was frequently heterogeneous, showing a bimodal pattern in 50% of PDOs which has not been observed in cancer cell lines, and this phenomenon was associated with poor susceptibility to cibisatamab treatment.

### CEA expression plasticity in CRC PDOs

**Fig. 4:**
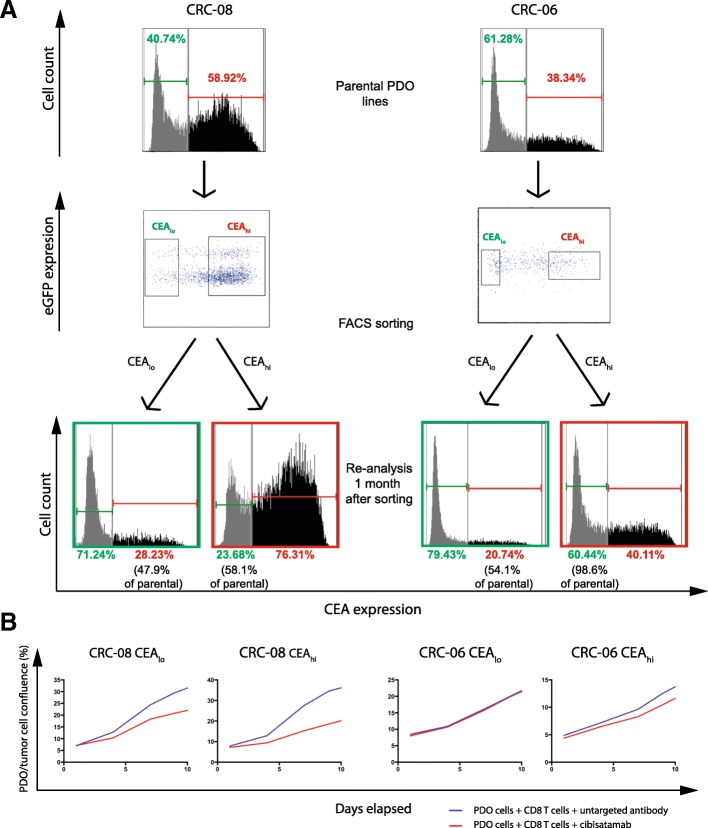
**a**: Baseline CEA expression profile measured by FACS, gates for sorting of CEA_lo_ and CEA_hi_ cells and CEA expression profiles of the sorted cells following re-expansion for 1 month. Sorting dot-blots also show GFP positivity of the PDO lines including a subpopulation of genome doubled cancer cells, that shows increased GFP signal due to higher histone content, which was repeatedly observed in CRC-08. **b**: Growth curves of sorted CEA_hi_ and CEA_lo_ PDO cells that had been re-expanded for 1 month before cibisatamab sensitivity was re-analysed in the PDO and T cell co-culture assay at an E:T ratio of 2:1

We next assessed if the CEA_hi_ and CEA_lo_ populations in PDOs were stable subpopulations or if CEA expression would change over time. CEA_hi_ and CEA_lo_ cells were FACS sorted from CRC-08 and CRC-06. Cells with intermediate expression levels were discarded during sorting to assure that only cells with very low and high CEA expression were included (Fig. 4a). CEA_hi_ and CEA_lo_ populations were then re-expanded for 1 month before CEA expression profiling and killing assays were repeated.

The sorted CEA_lo_ cells had each re-established CEA_hi_ subpopulations which were between 47.9 and 54.1% of the size of the CEA_hi_ population in the parental PDOs (Fig. 4a). Sorted CEA_hi_ cells also re-established CEA_lo_ cell populations but with noticeable different conversion rates in the two PDOs. CEA_hi_ cells from CRC-08 had re-generated 58.1% of the CEA_lo_ population size present in the parental cells whereas those from PDO CRC-06, which harbored a much larger proportion of CEA_lo_ cells in the parental population than CRC-08, had re-generated a CEA_lo_ population that was 98.6% of the size of the parental CEA_lo_ population. This demonstrates a high level of plasticity of CEA expression which rapidly re-establishes a heterogeneous population.

We next re-analysed the sensitivity to cibisatamab and CD8 T cell treatment in the cells that had been re-expanded from sorted CEA_hi_ and CEA_lo_ cells. Similar to the results obtained in parental PDOs with mixed CEA expression, treatment with cibisatamab had low efficacy (Fig. 4b), further corroborating that CEA_lo_ cells confer resistance. This experiment shows that CEA expression is highly plastic in many PDOs which promotes population heterogeneity and cibisatamab resistance.

### Identification of pathways regulating CEA expression

**Fig. 5:**
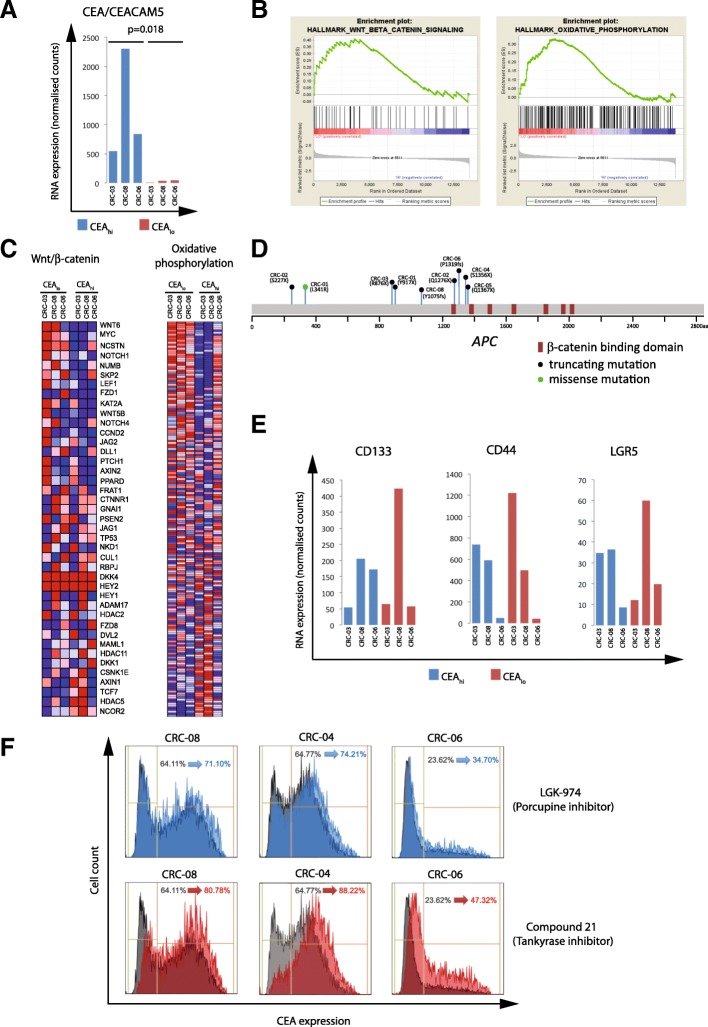
**a**: CEA/CEACAM5 mRNA expression in sorted CEA_hi/lo_ cells. A paired Student’s t-test was applied to log2 transformed gene expression data for significance analysis. **b**: Significant results of the gene set enrichment analysis of sorted CEA_hi_ and CEA_lo_ cells from CRC-03, CRC-08 and CRC-06. WNT/β-catenin and oxidative phosphorylation signatures were significantly enriched in cells expressing low CEA levels. **c**: mRNA expression heatmaps generated by GSEA showing expression levels of genes in the significantly enriched pathways. **d**: Representation of the APC gene and of the specific protein changes encoded by APC mutations found in 7/8 PDOs. **e**: Expression of genes which have been described as CRC stem cell markers in sorted CEA_hi/lo_ cells. **f**: CEA expression analysis of PDOs treated for 4 days with DMSO control (grey), with a 10uM of porcupine inhibitor (blue) or 10uM of a Tankyrase inhibitor

We flow sorted CEA_hi_ and CEA_lo_ cells from 3 PDOs and performed RNA expression analysis to investigate the mechanisms that regulate CEA expression and generate heterogeneity (Additional file 1: Table S1). The median expression of CEACAM5 mRNA, which encodes for the CEA protein, was 22.8 times higher in the sorted CEA_hi_ cells compared to the CEA_lo_ cells (*p* = 0.018; paired t-test; Fig. 5a), demonstrating that CEA surface expression heterogeneity in these PDOs is regulated at the gene expression level. We next applied gene set enrichment analysis (GSEA) [18] to identify potential molecular pathways which associate with CEA gene expression levels. Oxidative phosphorylation and WNT/β-catenin signalling were the only significantly enriched signatures following multiple testing correction and both were upregulated in the CEA_lo_ populations (Fig. 5b and c).

Our aim was to identify regulatory and potentially actionable pathways that control CEA expression and we hence focused subsequent analyses on the WNT/β-catenin signalling pathway. This pathway is genetically activated in the majority of CRCs, most frequently through mutations and loss of heterozygosity of the *APC* tumor suppressor gene and less commonly through mutations of other regulators of WNT signalling such as *RNF43* or in *β-catenin/CTNNB1* itself [21, 22]. Seven of the PDO samples harbored truncating mutations in the *APC* gene which result in gene products lacking six or seven of the seven β-catenin binding domains in the APC gene (Fig. 5d). Two independent *APC* mutations were identified in each of two PDOs (CRC-01, CRC-02). All three PDOs in which GSEA analysis had shown different WNT/β-catenin signatures activity in FACS sorted CEA_hi_ and CEA_lo_ subpopulations harbored *APC* mutations. This demonstrated that the WNT pathway remains regulated in these PDOs despite the presence of *APC* mutations, a phenomenon previously observed in CRC model systems and patient tumors [23–25]. Moreover, there was no association between the number of β-catenin binding domains that were inactivated by APC mutations and the fraction of CEA_lo_ and CEA_hi_ cells in these eight PDOs.

High WNT/β-catenin pathway activity and absence of CEA expression are features of the intestinal crypt bottom where intestinal stem cells reside [26] [27]. ,Moreover, high WNT/β-catenin pathway activity is also a characteristic of colon cancer stem cells [28]. However, the stem cell markers LGR5, CD133 or CD44 were not upregulated in CEA_lo_ compared to CEA_hi_ cells (Fig. 5e), suggesting that CEA_lo_ cells do not represent a classical CRC stem cell population [29].

### WNT/β-catenin pathway inhibition increases CEA expression

We finally investigated if pharmacological inhibition of the WNT/β-catenin pathway enhances CEA expression as predicted by these data. Three PDO lines with mixed CEA expression were treated with two different inhibitors of WNT signalling: a) the porcupine inhibitor LGK-974 which prevents WNT ligand secretion and hence autocrine and paracrine WNT-receptor activation and b) the tankyrase inhibitor compound 21 which inhibits the downstream WNT/β-catenin pathway by stabilizing the β-catenin destruction complex [17, 30]. Both compounds increased CEA expression and the CEA_hi_ subpopulation in all three PDOs (Fig. 5f). The tankyrase inhibitor consistently led to a stronger increase of CEA expression than porcupine inhibitor treatment, perhaps suggesting a limited contribution of auto- or paracrine WNT ligands to pathway regulation in these cells. These results confirmed a role of WNT/β-catenin signalling as a regulator of CEA expression in CRC PDOs.

## Discussion

This analysis of cibisatamab immunotherapy in a co-culture model of allogeneic T cells and PDOs, established mainly from metastatic chemotherapy resistant CRCs, confirmed high cell surface CEA expression as a key determinant of treatment sensitivity in vitro. Importantly, in contrast to cell lines, we found that CEA expression is frequently heterogeneous within PDO populations, resulting in bimodal CEA expression patterns in its most extreme form. We furthermore demonstrated high phenotypic plasticity that enables CRC cells to switch between high and low CEA expression. These results are likely highly relevant for CEA targeting immunotherapies as all 4/8 PDOs with mixed CEA expression profiles were at least partially resistant to cibisatamab treatment in vitro.

The observation that FACS sorted CEA_hi_ cells from the predominantly CEA_lo_ PDO CRC-06 more rapidly converted back to the CEA_lo_ state than those from the CEA_mixed_ PDO CRC-08 furthermore suggest that differences in the transition rate from high to low CEA and vice versa may determine the abundance of CEA_hi_ and CEA_lo_ cells at the long term equilibrium. Moreover, gene expression profiles of CEA_hi_ and CEA_lo_ cells from individual PDOs demonstrated that cell surface expression of CEA is regulated at the gene expression level and negatively correlates with WNT/β-catenin pathway activity. CEA expression is tightly regulated in healthy colonic crypts where expression is absent at the crypt bottom that harbors intestinal stem cells and gradually *increases* in epithelial cells as they become more differentiated towards the top of the crypt. The activity of WNT/β-catenin signalling physiologically *decreases* along the same axis, from the highest levels at the crypt bottom to low signalling activity at the top of the crypt. Together with our results that demonstrate a negative correlation between CEA expression and a WNT/β-catenin activity signature in PDOs, this suggests that the regulation of CEA expression heterogeneity in PDOs and in healthy colon epithelial cells is similar.

We found no correlation between the degree of *APC* protein truncation by distinct mutations, which is likely to lead to different levels of β-catenin signalling pathway activation [25], with CEA expression levels or CEA heterogeneity. In addition, we showed that β-catenin signalling remains regulated in PDOs despite genetic activation through APC mutations. Preservation of WNT/β-catenin pathway regulation and heterogeneity despite genetic activation of the pathway have recently been shown in CRC cell lines and animal models and this has been attributed to a several cell intrinsic, autocrine and also stromal factors that influence multiple pathways converging on b-catenin [23–25]. Similar mechanisms may explain heterogeneity in these PDO models.

The upregulation of a signature of oxidative phosphorylation in CEA_lo_ cells highlights further similarities to intestinal stem cells at the crypt bottom which are more dependent on oxidative phosphorylation than their more differentiated progeny towards the top of the crypt [31]. Despite the gene expression similarities between CEO_lo_ cells in PDOs and the crypt bottom where stem cells reside, specific genes which have frequently been associated with stemness in CRC were not upregulated in the CEO_lo_ cells from our PDOs. This may indicate a dissociation of the mechanisms that regulate differentiation processes including CEA expression and stemness in PDOs from advanced and drug resistant tumours but this requires further investigation.

The critical role of CEA expression heterogeneity and plasticity as determinants of treatment resistance in our in vitro models may be relevant for the characterization of predictive biomarkers for CEA-targeting immunotherapies in CRC where heterogeneous CEA expression has been described. Moreover, finding strategies to increase CEA expression may increase the clinical responses of cibisatamab.

PDOs harboring predominantly CEA_hi_ cells were highly sensitive to cibisatamab treatment in vitro but the low level of expression plasticity we observed may nevertheless enable gradual loss of the target antigen during prolonged treatment. The plasticity of CEA expression which re-establishes a CEA_hi_ population after FACS sorting of CEA_lo_ cells suggests that re-treatment with cibisatamab after a prolonged treatment break may lead to further responses. Phenotypic plasticity may furthermore be amenable to combination therapies which either increase CEA expression in the CEA_lo_ population or which co-target vulnerabilities of the CEA_lo_ subpopulations while also administering cibisatamab. Our results provided proof of principle that CEA expression can be pharmacologically perturbed and indicate WNT pathway inhibitors, which are in clinical development, as a potential strategy to boost CEA expression and increase therapeutic benefit from bispecific CEA-targeting antibodies. However, the WNT pathway has an essential role in the development and the homeostasis of many normal tissues in the human body. As a consequence, targeting of the WNT pathway has been challenging in preclinical models and clinical trials, particularly due to gastrointestinal toxicities [32]. However, there is cautious optimism that a better understanding of WNT regulation in malignant cells and of the cross-talk between the WNT and other signaling pathways in cancer may enable the design of new molecules with a broader therapeutic window and lower toxicity [30]. Our results also question whether CEA_lo_ cells may be susceptible to inhibition of oxidative phosphorylation as an alternative strategy to address this subpopulation. Several CEA targeting immunotherapies beyond bispecific T cell redirecting antibodies are currently in preclinical or clinical development, including CEA vaccines and CEA targeting CAR T cells or TCR engineered T cells [33]. CEA expression plasticity and heterogeneity are likely hindrances for each of these approaches. Together with the limited success that has so far achieved in MSS CRCs with checkpoint inhibiting immunotherapies alone, strategies to enhance CEA expression in combination with CEA targeting therapies appear promising to extend immunotherapy benefit to a larger proportion of CRC patients.

Finally, this study shows the potential of PDO and allogeneic T cell co-cultures as novel tools to dissect mechanisms of immunotherapy resistance in vitro*.* This addresses a major need in translational immunotherapy research, particularly as studies in immunocompetent animal models are often slow and expensive and sometimes even impossible as animal models are not available for all tumor types, or are incompatible with human-specific reagents, including many monoclonal antibodies. Allogeneic systems can be hampered by alloreactivity where donor T cells recognize MHC molecules on target cells as non-self. This can lead to high background killing activity that may impair assay sensitivity and specificity compared to autologous systems. Cancer cells alone as well as in co-culture with T cells but without antibody were included as controls in our assays. This allowed us to identify and exclude the small number of experiments in which alloreactivity impaired cancer cell growth. Using allogeneic T cells in PDO co-culture models hence represent a valid alternative strategy when autologous T cells are unavailable. These PDO T cell co-culture models can accelerate the discovery of immune-oncology candidate resistance mechanisms, biomarkers of efficacy and define rational combination therapy strategies for clinical testing. The ability of PDOs to represent intra-tumor heterogeneity to some degree, and the opportunity to establish them from tumors which match the stage and the treatment history of patients in which novel drugs are tested, are decisive advantages for pre-clinical discovery.

## Conclusions

CEA expression heterogeneity is common in CRC PDOs from therapy resistant metastatic CRCs and this confers cibisatamab resistance in vitro. Whether CEA expression heterogeneity is associated with cibisatamab resistance should be investigated in clinical trial samples. CEA expression can be pharmacologically enhanced through WNT/β-catenin pathway inhibition, suggesting opportunities for combination therapies that should increase cibisatamab efficacy in the clinic.

## Additional file

Additional file 1: **Table S1.** Genome-wide RNA expression analysis of FACS sorted CEAhi and CEAlo PDO cells. (XLSX 1049 kb)

## Abbreviations

CAR: Chimeric Antigen Receptor
CEA: Carcino Embryonic Antigen
CEA-TCB: Carcino Embryonic Antigen-T Cell Bispecific
CRC: Colorectal cancer
E:T: Effector to Target
eGFP: enhance Green Fluorescent Protein
FACS: Fluorescence-Activated Cell Scanning
GSEA: Gene Set Enrichment Analysis
IL2: Interleukin 2
MFI: Mean Fluorescence Intensity
MSI: Microsatellite instable
MSS: Microsatellite stable
PBMCs: Peripheral Blood Mononuclear Cells
PD-L1: Programmed Death Ligand 1
PDO: Patient Derived Organoid
TCR: T Cell Receptor

## References

[1] Siegel RL, Miller KD, Fedewa SA, Ahnen DJ, Meester RGS, Barzi A (2017). Colorectal Cancer statistics , 2017. CA Cancer J Clin.

[2] Van Cutsem E, Cervantes A, Nordlinger B, Arnold D (2014). Metastatic colorectal cancer: ESMO clinical practice guidelines for diagnosis, treatment and follow-up. Ann Oncol.

[3] Mayer RJ, Van Cutsem E, Falcone A, Yoshino T, Garcia-Carbonero R, Mizunuma N (2015). Randomized trial of TAS-102 for refractory metastatic colorectal Cancer. N Engl J Med.

[4] Grothey A, Van CE, Sobrero A, Siena S, Falcone A, Ychou M (2013). Regorafenib monotherapy for previously treated metastatic colorectal cancer (CORRECT): an international, multicentre, randomised, placebo-controlled, phase 3 trial. Lancet..

[5] Le DT, Uram JN, Wang H, Bartlett BR, Kemberling H, Eyring AD (2016). HHS public access. N Engl J Med.

[6] Overman MJ, McDermott R, Leach JL, Lonardi S, Lenz H-J, Morse MA, et al. Nivolumab in patients with metastatic DNA mismatch repair-deficient or microsatellite instability-high colorectal cancer (CheckMate 142): an open-label, multicentre, phase 2 study. Lancet Oncol 2017/07/19. 2017 Sep;18(9):1182–1191. 10.1016/S1470-2045(17)30422-9 PMC6207072 28734759

[7] Llosa NJ, Cruise M, Tam A, Wicks EC, Hechenbleikner EM, Taube JM (2015). The vigorous immune microenvironment of microsatellite instable colon cancer is balanced by multiple counter-inhibitory checkpoints. Cancer Discov.

[8] Koopman M, Kortman G A M, Mekenkamp L, Ligtenberg M J L, Hoogerbrugge N, Antonini N F, Punt C J A, van Krieken J H J M (2009). Deficient mismatch repair system in patients with sporadic advanced colorectal cancer. British Journal of Cancer.

[9] Chau I (2017). Clinical development of PD-1/PD-L1 immunotherapy for gastrointestinal cancers: facts and hopes. Clin Cancer Res.

[10] Bacac M, Klein C, Umana P (2016). CEA TCB: a novel head-to-tail 2:1 T cell bispecific antibody for treatment of CEA-positive solid tumors. Oncoimmunology..

[11] Bacac M, Fauti T, Sam J, Colombetti S, Weinzierl T, Ouaret D (2016). A novel carcinoembryonic antigen T-cell bispecific antibody (CEA TCB) for the treatment of solid tumors. Clin Cancer Res.

[12] Argilés G, Saro J, Segal NH, Melero I, Ros W, Marabelle A (2017). LBA-004Novel carcinoembryonic antigen T-cell bispecific (CEA-TCB) antibody: preliminary clinical data as a single agent and in combination with atezolizumab in patients with metastatic colorectal cancer (mCRC). Ann Oncol.

[13] Tabernero J, Melero I, Ros W, Argiles G, Marabelle A, Rodriguez-Ruiz ME (2017). Phase Ia and Ib studies of the novel carcinoembryonic antigen (CEA) T-cell bispecific (CEA CD3 TCB) antibody as a single agent and in combination with atezolizumab: preliminary efficacy and safety in patients with metastatic colorectal cancer (mCRC). J Clin Oncol.

[14] Sato T, Stange DE, Ferrante M, Vries RGJ, Van Es JH, Van Den Brink S (2011). Long-term expansion of epithelial organoids from human colon, adenoma, adenocarcinoma, and Barrett’s epithelium. Gastroenterology..

[15] Beronja Slobodan, Livshits Geulah, Williams Scott, Fuchs Elaine (2010). Rapid functional dissection of genetic networks via tissue-specific transduction and RNAi in mouse embryos. Nature Medicine.

[16] Love MI, Huber W, Anders S. Moderated estimation of fold change and dispersion for RNA-seq data with DESeq2. Genome Biol. 2014/12/05. 2014;15(12):550. 10.1186/s13059-014-0550-8 PMC4302049 25516281

[17] Elliott RJR, Jarvis A, Rajasekaran MB, Menon M, Bowers L, Boffey R (2015). Design and discovery of 3-aryl-5-substituted-isoquinolin-1-ones as potent tankyrase inhibitors. Medchemcomm..

[18] Subramanian A., Tamayo P., Mootha V. K., Mukherjee S., Ebert B. L., Gillette M. A., Paulovich A., Pomeroy S. L., Golub T. R., Lander E. S., Mesirov J. P. (2005). Gene set enrichment analysis: A knowledge-based approach for interpreting genome-wide expression profiles. Proceedings of the National Academy of Sciences.

[19] Yan C, Hu Y, Zhang B, Mu L, Huang K, Zhao H (2016). The CEA−/lo colorectal cancer cell population harbors cancer stem cells and metastatic cells. Oncotarget..

[20] Uhlén M, Fagerberg L, Hallström BM, Lindskog C, Oksvold P, Mardinoglu A, et al. Tissue-based map of the human proteome. Science. (80- ). 2015 Jan 23;347(6220). 10.1126/science.1260419 25613900

[21] Network CGA (2012). Comprehensive molecular characterization of human colon and rectal cancer. Nature..

[22] Giannakis Marios, Hodis Eran, Jasmine Mu Xinmeng, Yamauchi Mai, Rosenbluh Joseph, Cibulskis Kristian, Saksena Gordon, Lawrence Michael S, Qian Zhi Rong, Nishihara Reiko, Van Allen Eliezer M, Hahn William C, Gabriel Stacey B, Lander Eric S, Getz Gad, Ogino Shuji, Fuchs Charles S, Garraway Levi A (2014). RNF43 is frequently mutated in colorectal and endometrial cancers. Nature Genetics.

[23] Brabletz T, Jung A, Reu S, Porzner M, Hlubek F, Kunz-Schughart LA (2001). Variable beta-catenin expression in colorectal cancers indicates tumor progression driven by the tumor environment. Proc Natl Acad Sci U S A.

[24] Vermeulen L, De Sousa E, Melo F, van der Heijden M, Cameron K, de Jong JH, Borovski T (2010). Wnt activity defines colon cancer stem cells and is regulated by the microenvironment. Nat Cell Biol.

[25] Voloshanenko O, Erdmann G, Dubash TD, Augustin I, Metzig M, Moffa G (2013). Wnt secretion is required to maintain high levels of Wnt activity in colon cancer cells. Nat Commun.

[26] Jothy S, Yuan SY, Shirota K (1993). Transcription of carcinoembryonic antigen in normal colon and colon carcinoma. In situ hybridization study and implication for a new in vivo functional model. Am J Pathol.

[27] Barker N (2013). Adult intestinal stem cells: critical drivers of epithelial homeostasis and regeneration. Nat Rev Mol Cell Biol.

[28] de Sousa EMF, Vermeulen L, Richel D, Medema JP (2011). Targeting Wnt signaling in Colon Cancer stem cells. Clin Cancer Res.

[29] Vermeulen L, Snippert HJ (2014). Stem cell dynamics in homeostasis and cancer of the intestine. Nat Rev Cancer.

[30] Mariotti L, Pollock K, Guettler S (2017). Regulation of Wnt/β-catenin signalling by tankyrase-dependent poly(ADP-ribosyl)ation and scaffolding. Br J Pharmacol.

[31] Rodríguez-Colman MJ, Schewe M, Meerlo M, Stigter E, Gerrits J, Pras-Raves M (2017). Interplay between metabolic identities in the intestinal crypt supports stem cell function. Nature..

[32] Kahn M (2014). Can we safely target the WNT pathway?. Nat Rev Drug Discov.

[33] Parkhurst MR, Yang JC, Langan RC, Dudley ME, Nathan D-AN, Feldman SA, et al. T cells targeting carcinoembryonic antigen can mediate regression of metastatic colorectal cancer but induce severe transient colitis. Mol Ther 2010/12/14. 2011 Mar. 19(3):620–6. 10.1038/mt.2010.272 PMC3048186 21157437

